# Benefits of Higher Cardiovascular and Motor Coordinative Fitness on Driving Behavior Are Mediated by Cognitive Functioning: A Path Analysis

**DOI:** 10.3389/fnagi.2021.686499

**Published:** 2021-06-29

**Authors:** Robert Stojan, Navin Kaushal, Otmar Leo Bock, Nicole Hudl, Claudia Voelcker-Rehage

**Affiliations:** ^1^Institute of Sport and Exercise Sciences, University of Muenster, Muenster, Germany; ^2^Institute of Human Movement Science and Health, Chemnitz University of Technology, Chemnitz, Germany; ^3^School of Health & Human Sciences, Indiana University, Bloomington, IA, United States; ^4^Institute of Exercise Training and Sport Informatics, German Sport University Cologne, Cologne, Germany

**Keywords:** aging, car driving, dual-tasking, multitasking, executive functions, fitness, virtual reality, ecological validity

## Abstract

Driving is an important skill for older adults to maintain an independent lifestyle, and to preserve the quality of life. However, the ability to drive safely in older adults can be compromised by age-related cognitive decline. Performing an additional task during driving (e.g., adjusting the radio) increases cognitive demands and thus might additionally impair driving performance. Cognitive functioning has been shown to be positively related to physical activity/fitness such as cardiovascular and motor coordinative fitness. As such, a higher fitness level might be associated with higher cognitive resources and may therefore benefit driving performance under dual-task conditions. For the first time, the present study investigated whether this association of physical fitness and cognitive functioning causes an indirect relationship between physical fitness and dual-task driving performance through cognitive functions. Data from 120 healthy older adults (age: 69.56 ± 3.62, 53 female) were analyzed. Participants completed tests on cardiovascular fitness (cardiorespiratory capacity), motor coordinative fitness (composite score: static balance, psychomotor speed, bimanual dexterity), and cognitive functions (updating, inhibition, shifting, cognitive processing speed). Further, they performed a virtual car driving scenario where they additionally engaged in cognitively demanding tasks that were modeled after typical real-life activities during driving (typing or reasoning). Structural equation modeling (path analysis) was used to investigate whether cardiovascular and motor coordinative fitness were indirectly associated with lane keeping (i.e., variability in lateral position) and speed control (i.e., average velocity) while dual-task driving *via* cognitive functions. Both cardiovascular and motor coordinative fitness demonstrated the hypothesized indirect effects on dual-task driving. Motor coordinative fitness showed a significant indirect effect on lane keeping, while cardiovascular fitness demonstrated a trend-level indirect effect on speed control. Moreover, both fitness domains were positively related to different cognitive functions (processing speed and/or updating), and cognitive functions (updating or inhibition), in turn, were related to dual-task driving. These findings indicate that cognitive benefits associated with higher fitness may facilitate driving performance. Given that driving with lower cognitive capacity can result in serious consequences, this study emphasizes the importance for older adults to engage in a physically active lifestyle as it might serve as a preventive measure for driving safety.

## Introduction

Driving a car is an essential skill for older adults to preserve mobility and independent living (Owsley, 2002; Musselwhite et al., 2015). It has been suggested previously (Anstey et al., 2005; Karthaus and Falkenstein, 2016) that controlling a vehicle affords an effective integration of multiple perceptual (e.g., visual information), motor (e.g., upper and lower limb control), and cognitive functions (e.g., visuospatial skill, attention, cognitive processing speed). Those functions usually decline with higher age (Schaie and Willis, 2010; Seidler et al., 2010; Roberts and Allen, 2016; Anderson and Craik, 2017). As a result, car driving becomes increasingly cognitively demanding for older adults (Karthaus and Falkenstein, 2016). Cognitive demand during driving further increases when an additional task is performed concomitantly (i.e., dual- or multitasking), such as when adjusting the radio or talking to passengers (Vernon et al., 2015; Young et al., 2017; Depestele et al., 2020). In these complex, cognitively demanding situations, older adults are particularly at risk for accidents (Owsley et al., 1991; Aschersleben and Müsseler, 2008; Bélanger et al., 2010, 2015; Klauer et al., 2014; Dingus et al., 2016; Lombardi et al., 2017) as they seem to have difficulties in distributing their cognitive resources to both tasks simultaneously (Verhaeghen et al., 2003; Brustio et al., 2017). A higher level of cognitive functioning in older adults has been shown to be positively associated with their physical fitness level, both cardiovascular fitness and motor coordinative fitness (Voelcker-Rehage et al., 2010; Voelcker-Rehage and Niemann, 2013; Freudenberger et al., 2016). Hence being physically active/fit might not only preserve cognitive functioning but also driving performance, particularly under cognitively demanding conditions. No study, however, has yet investigated whether driving in the presence of an additional cognitive demand, as it is typically the case in daily life situations, indirectly benefits from higher physical fitness through higher cognitive functioning. Here, we address this issue using structural equation modeling (path analysis).

Age-related cognitive decline affects several cognitive functions that are associated with driving behavior such as attention, visuospatial skill, memory, or cognitive processing speed (Verhaeghen et al., 2003; Apolinario et al., 2009; Gajewski and Falkenstein, 2011; Wasylyshyn et al., 2011; Young and Bunce, 2011; Harada et al., 2013; Murman, 2015; Fraade-Blanar et al., 2018; Salthouse, 2019). For example, both visuospatial skill and attention are required to continuously monitor the environment while being able to very quickly identifying potential hazards on the road (Andrews and Westerman, 2012; Michaels et al., 2017; Eudave et al., 2018; Ledger et al., 2019). Many studies have demonstrated such positive associations between different cognitive functions and driving behavior in a variety of scenarios (e.g., car following, braking, overtaking), and for different performance parameters (e.g., lane keeping, speed control, braking reactions; Young and Bunce, 2011; Anstey et al., 2012; Depestele et al., 2020). Interestingly, the involvement of cognitive functions during driving seems to be more pronounced in older than in younger adults (Anstey et al., 2005; Lees et al., 2010; Fraade-Blanar et al., 2018), even though older adults are usually more experienced drivers and might preserve a relatively high level of automatization in driving (McKenna and Farrand, 1999; Lees et al., 2010; Charlton and Starkey, 2011; Anstey et al., 2012). When engaging in more complex or unexpected and hazardous situations, fluid cognitive functions such as inhibition, updating, shifting, and cognitive processing speed seem to be required in particular, and especially in older persons (Young and Bunce, 2011; Karthaus and Falkenstein, 2016). The former three functions (inhibition, updating, shifting) are often summarized under the umbrella term “executive functions” (see Miyake et al., 2000; Miyake and Friedman, 2012; Bock et al., 2019b). More recently, these functions have been discussed to play an important role in driving and accident risk among older adults, and particularly when simultaneously being involved in a cognitively demanding task (Mathias and Lucas, 2009; Asimakopulos et al., 2012; Harada et al., 2013; Karthaus and Falkenstein, 2016; Eramudugolla et al., 2017; Walshe et al., 2017; Haeger et al., 2018). For example, *inhibiting non-relevant or distracting information* during car driving is essential to keep attention focused on the road. In addition, when engaging in an additional task during driving (e.g., adjusting the radio) the driver is required to efficiently *shift* attention *between tasks* while also *holding and updating relevant environmental cues in mind* (Marmeleira et al., 2009; Karthaus and Falkenstein, 2016; Pope et al., 2017). Further, *cognitive processing speed* has been associated with driving behavior (Salthouse, 1996, 2009; Roenker et al., 2003; Edwards et al., 2009; Albinet et al., 2012; Eramudugolla et al., 2017), as it is important during complex cognitive-motor behaviors such as high traffic loads or driving while performing an additional task (Edwards et al., 2009; Künstler et al., 2018; Andersson and Peters, 2020). However, the cognitive mechanisms specifically involved in dual-task driving need to be further investigated as most studies on the relationship between cognitive functions and driving have been conducted in single-task settings (Depestele et al., 2020).

The level of cognitive functioning in older adults seems to be influenced by several lifestyle factors (Reuter-Lorenz and Park, 2014). In particular physical activity/fitness, such as cardiovascular and motor coordinative fitness, are positively related to cognitive functioning (Voelcker-Rehage and Niemann, 2013; Levin et al., 2017; Cabeza et al., 2018; Nystoriak and Bhatnagar, 2018; James et al., 2019). A large body of cross-sectional and longitudinal studies demonstrates this relationship (Voelcker-Rehage et al., 2010; Bherer et al., 2013; Diamond, 2013; Bherer, 2015; Dupuy et al., 2015; Young et al., 2015; Gajewski and Falkenstein, 2016; Gheysen et al., 2018; Diamond and Ling, 2019; Hillman et al., 2019; Ludyga et al., 2020), particularly for executive functions (Colcombe and Kramer, 2003; Angevaren et al., 2008; Voelcker-Rehage et al., 2011; Park and Bischof, 2013; Kaushal et al., 2018; Stojan and Voelcker-Rehage, 2019). These benefits have been attributed to numerous and overlapping neurobiological adaptations, including higher gray matter volume (Voelcker-Rehage and Niemann, 2013; Erickson et al., 2014) and preserved white matter structure (Tseng et al., 2013; Sexton et al., 2016; Kim et al., 2020), increased cerebral blood flow and vascularization (Sonntag et al., 2007; Tarumi and Zhang, 2018; Bliss et al., 2021), as well as improved connectivity between brain regions (Voss et al., 2013, 2015). Executive functions might particularly benefit from these structural and functional brain changes as they depend on a distributed neural network across different brain regions (Niendam et al., 2012; Diamond, 2013). Especially (pre)frontal areas, but also other brain regions in the parietal and temporal cortex, are associated with executive functions (Alvarez and Emory, 2006; Shokri-Kojori et al., 2012). Even though these brain areas are quite vulnerable to the effects of age, they are also sensitive to the beneficial effects of physical fitness/activity (Bherer et al., 2013; Gomez-Pinilla and Hillman, 2013; Voelcker-Rehage and Niemann, 2013). Thus, higher physical fitness/activity may facilitate the neural basis of executive functioning in particular. In addition, executive functions also seem to profit from higher cognitive processing speed. Cognitive processing speed is also largely associated with white matter structure (Kerchner et al., 2012; Kuznetsova et al., 2016). As such, well-preserved white matter integrity and myelination enable faster signal transmission, thus further strengthening the effective integration of executive functions. These described effects of physical activity/fitness on cognitive and brain function, however, seem to differ not only with respect to the cognitive dimension but also with respect to the physical dimension (Colcombe and Kramer, 2003; Voelcker-Rehage et al., 2011; Voelcker-Rehage and Niemann, 2013; Barha et al., 2017; Ludyga et al., 2020). As such, cardiovascular and motor coordinative fitness have been attributed to distinct neurobiological mechanisms that seem to promote specific cognitive functions (Black et al., 1990; Voelcker-Rehage and Niemann, 2013; Tarumi and Zhang, 2018; Walsh and Tschakovsky, 2018; Walsh et al., 2020). Therefore, associations between physical fitness and specific cognitive functions often vary across studies and yet have to be investigated more systematically.

Put together, the extant literature suggests that driving behavior depends on fluid cognitive functions and that cognitive demands increase when executing an additional task during driving. The same fluid cognitive functions that are required for driving might benefit from cardiovascular and/or motor coordinative fitness in older adults. Therefore, the association of physical fitness with fluid cognitive functions may establish a positive indirect relationship between fitness and driving performance, particularly in cognitively demanding conditions (e.g., during dual-tasking). Higher cardiovascular and motor coordinative fitness, thus, may indirectly facilitate driving performance through higher cognitive functioning. Direct effects of physical fitness on driving, in turn, seem less conceivable, as driving a car is a physically low-demanding, sedentary behavior (with or without an additional task). Therefore, while previous studies only have focused on direct relationships between the described variables, in the current study we focused specifically on determining the described indirect relationships.

**Figure 1:**
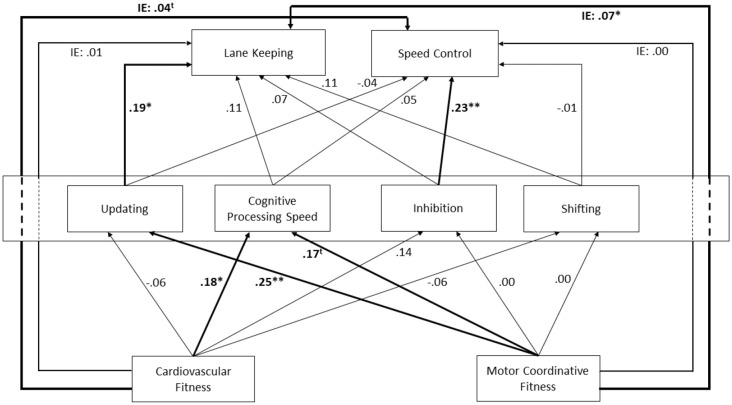
Path diagram on the relationships between fitness domains (lower part), cognitive functions (intermediate part) and driving behavior (upper part). Bold arrows indicate significant (*p* < 0.05) or trend level effects (*p* < 0.10). Outer dotted lines indicate indirect effects (labeled ‘IE’) through cognitive functions. Note: *p < 0.05; **p < 0.01; ^t^*p* < 0.10.

In this study, we hence aimed to examine the additional benefits that higher cardiovascular and/or motor coordinative fitness potentially yield for car driving performance in cognitively demanding conditions by mediation through cognitive functions. To mimic the varying cognitive demands of everyday car driving, a realistic virtual driving scenario that included a battery of additional tasks was implemented (see “Driving Simulator Setup and Scenario” section). The rationale for using a driving simulator was to provide a safe virtual environment in which participants can engage in complex and potentially hazardous (dual-task) situations while driving (Bock et al., 2018). In addition, virtual environments can be fully controlled regarding the implementation of additional tasks as well as environmental conditions such as ambient light, traffic sounds, other traffic participants, or weather (Bock et al., 2019a). A driving simulator, thus, ensured the same conditions for all participants. The experimental outcome was evaluated by path analysis to determine the complex relationships between driving, fluid cognitive functions, and physical fitness (for further information see “Statistical Approach” section). The primary outcomes of interest in driving were: (1) variability in lateral car position (Latpos SD) as an indicator of *lane keeping*, and (2) mean velocity (Velocity M) as an indicator of *speed control*. Both parameters are common measures of driving performance and were found to be particularly sensitive to driving under dual-tasking conditions in previous studies (Papantoniou et al., 2017; Wechsler et al., 2018; Depestele et al., 2020; Stojan and Voelcker-Rehage, 2021). Four fluid cognitive functions (inhibition, shifting, updating, cognitive processing speed) were assessed and set separately as intermediate dependent variables in our path model (see “Statistical Approach” section and Figure 1). In addition, two indicators of physical fitness were entered as separate independent variables into the model, i.e., cardiovascular fitness (cardiorespiratory capacity *via* peak oxygen consumption) and motor coordinative fitness (composite score of static balance, psychomotor speed, bimanual dexterity). We assumed differential associations between various cognitive functions and driving performance, and differential relationships of cardiovascular and motor coordinative fitness with cognitive functions. According to our main research question, we hypothesized a positive indirect effect of both cardiovascular and motor coordinative fitness on driving performance *via* indirect pathways through fluid cognitive functions. Furthermore, we also expected distinct indirect effects of cardiovascular and motor coordinative fitness on driving performance based on their differential neurobiological mechanisms and relationships with cognitive functions. This is the first study to examine the indirect benefits of physical activity/fitness on dual-task driving in healthy older adults.

## Materials and Methods

### Study Design and Participants

For the purpose of this study, data from two phases of a larger project (from here on referred to as project phase I and project phase II) were combined to ensure sufficient statistical power for the proposed model. Both data sets were collected as part of the DFG (German Research Foundation) Priority Program SPP 1772 “Multitasking”. Some data from project phase I have been already published elsewhere (Wechsler et al., 2018; Bock et al., 2019b), and other data will be published later on. In total, data from 120 healthy older adults between 64 and 79 years of age (*M* = 69.56 ± 3.62 years, *f* = 53) were pooled (sample 1, *n* = 61, sample 2, *n* = 59). Demographic characteristics of the two samples are presented in Table 1, indicating that both samples were comparable regarding age, sex distribution, education, body mass index (BMI), and cognitive status. Study designs, recruitment strategies, inclusion/exclusion criteria, and applied tests were largely similar between project phases. Therefore, the two samples of project phase I and II should be considered cohorts of the same population. Community-dwelling, healthy older adults were recruited *via* public advertising, including radio reports, newspaper articles, flyers, and senior college talks. Participants were screened for eligibility during a structured phone interview lasting for 10–15 min. All inclusion criteria were self-reported and comprised the following conditions: Aged between 65 and 75 years [minor exceptions for spouses, *n* = 5: aged 64 (*n* = 1), 78 (*n* = 1), and 79 (*n* = 3)], BMI < 30, absence of physical (e.g., cardiovascular or orthopedic conditions), neurocognitive (e.g., dementia or traumatic brain injuries), or psychological (e.g., depression or anxiety disorders) medical conditions, regular driving activities during the last 6 months (at least one time per week), being able to walk for at least 30 min without any assistance. In the sample of project phase I, left- and right-handers were included (*n* = 5 left-handers), while in the sample of project phase II left-handers were excluded (exception for spouses, *n* = 3 left-handers). All participants self-reported normal or corrected to normal vision and hearing. After the eligibility check, further screening tests were applied including the Freiburg Visual Acuity Test v. 3.9.0 (cut-off: 20/60; Keeffe et al., 2002) and Mini-Mental-State-Examination (cut-off: 27/30; Creavin et al., 2016). No participant had to be excluded based on these tests. Furthermore, all participants had to obtain medical clearance and consent from their practitioner/cardiologist to participate in this study (exercise electrocardiography). All participants received monetary compensation (15€ per testing day). The ethics committee of the German Sport University, Cologne, Germany approved project phase I (Nr.: 27/2015), and the ethics committee of the Chemnitz University of Technology, Germany approved project phase II (Nr.: V-280-17-CVR-Multitasking-29062018). Both project phases were conducted in accordance with the latest version of the Declaration of Helsinki (World Medical Association, 2013). All participants signed an informed consent statement before testing.

**Table 1:** Participants’ demographic information.

	project phase I, *n* = 61 *M* (*SD*) or *n*	project phase II, *n* = 59 *M* (*SD*) or *n*	*t or x^2^*	*p (uncor.)*
Age (years)	69.93 (2.95)	69.17 (4.20)	1.15	0.253
Female/Male (*n*)	23/38	30/29	2.10	0.147
Education (years)	15.62 (3.21)	15.77 (2.57)	−0.27	0.780
Height (in m)	1.72 (0.09)	1.69 (0.08)	2.39	0.019
Weight (in kg)	74.76 (10.80)	70.92 (9.18)	2.10	0.038
BMI (kg/m^2^)	25.08 (2.44)	24.91 (2.85)	0.36	0.719
MMSE (0–30)	29.15 (1.00)	29.21 (0.85)	−0.35	0.730

*Note. Means (*M*) and standard deviations (*SD* in parentheses) are presented. BMI, body mass index; MMSE, mini-mental state examination; Uncorrected *p*-values are presented*.

### Measures

#### Driving Simulator Setup and Scenario

Driving behavior was assessed using commercially available driving simulator hardware and software (Carnetsoft^®^ version 8.0, Groningen, The Netherlands). The driving simulator consisted of three 48′′ monitors (laterally angled at 45°) on regular desks with a horizontal field of view of 195° (for a graphical illustration of the setup, see Wechsler et al., 2018). A VW golf seat, Logitech G27 steering wheel (Logitech International S.A., Lausanne Switzerland), and gas and brake pedals were located at positions similar to a real car, and a conventional numeric keypad was mounted on the right side near the steering wheel. Numbers from 1 to 6 were visible on the keypad (two rows with three numbers), other keys were covered with black tape. A head set was used for task presentation and characteristic driving sounds. The seat and gas and brake pedals were individually adjustable to fit every participant’s comfortable driving position. Motion sickness was minimized by utilizing a research-grade simulator with wide-screen displays for smooth rendering of visual motion. The visual field around the displays was covered by black cloth to reduce perceptual conflicts between central and peripheral vision.

The driving scenario lasted about 25 min (25.7 km) and simulated a typical rural environment: a road that was slightly winding through a landscape consisting of grasslands, clouds, small trees, animal enclosures, hay rolls, construction sides, road signs, and gas stations. No intersections, traffic lights, cyclists, or pedestrians were included. Oncoming traffic comprised other cars and buses. Participants drove a VW Golf and followed a lead car. Another car followed at a reasonable distance behind the participant’s car. The lead car was programmed to drive at 70 km/h and slowed down slightly when the distance with the driver exceeded 100 m. Participants were instructed to drive as they normally would, and to follow the lead car at a reasonable distance with a speed of 70 km/h unless other speed limits (i.e., 40 km/h during braking tasks) were specified. They were not allowed to pass the lead car, and they were told that no cars will pass them. Ten braking sections were included in the driving environment. When reaching one of those sections, the lead car briefly braked: it slowed down to 40 km/h for about 6 s and then sped up again to 70 km/h. Braking sections, however, were not further considered in the present study, and they did not overlap with the additional tasks outlined below. If the participants’ car crashed (e.g., into the lead car or oncoming traffic, rarely a cow/tree), the front window shattered (including acoustic feedback) and the driver’s car was relocated between the rear and lead car. Participants practiced driving for 3–4 min (driving only) in the same environment used for data acquisition. They also practiced the additional tasks for 3–4 min (tasks only) while their car drove in autopilot mode in the same environment. Participants did not practice dual-task driving. Instructions on driving and additional tasks were provided verbally. All participants followed instructions correctly during practice trials and during data acquisition, without asking for repetitions or for slower speech. From this, we concluded that their language comprehension and hearing was not overly degraded.

During driving, participants executed different additional tasks. These tasks were modeled after typical real-life activities often performed during driving. To increase realism and to mimic the varying demands of everyday car driving, we provided different stimulus modalities (visual input on the windshield = in-vehicle display, auditory input *via* headphones = passengers, radio, or GPS), cognitive-motor task loads (i.e., baseline driving = no task, typing = dashboard operations, reasoning = conversation with passengers), and response modalities (typing = visuomotor responses, reasoning = verbal responses; Bock et al., 2018, 2019a). The number of trials (total trials *N* = 60) was equally distributed across the different task types and presentation modalities in both project phases. Tasks were scheduled in a mixed order and at irregular distance intervals. The driving scenario and the order and type of additional tasks was identical for all participants within each project phase (same seed; Bock et al., 2019a). Participants were instructed not to prioritize the driving or the additional task, but to respond as fast and as accurately as possible to the additional task. The following tasks were utilized:

The* reasoning task* required participants to verbally state an argument for or against an issue of general interest (e.g., “state an argument against using electric cars”, in German language). Requests were limited to 10 words per sentence (max. 80 characters, 54 pt. font size, max. two lines) and could not be simply answered with “yes” or “no”. The visual presentation lasted 5 s, auditory presentation varied between 3 and 4 s. Participants were instructed to respond verbally while continuing to drive. Answers were assessed as valid/not valid and protocolled by the experimenter. The *typing task* required participants to enter a 3-digit number (e.g., “345”) into the numeric keypad to the right of the steering wheel. Only numbers consisting of the digits 1–6 were presented, and only those digits were accessible on the keypad. The visual presentation lasted 5 s, auditory presentation lasted about 3 s. The numbers entered and reaction times for each number were recorded digitally by the software.

Only in project phase I an additional memorizing task was used that was presented similar to the two tasks described above. Participants had to memorize and compare gas station prices (visual) and traffic news (auditory), respectively. In project phase II the memorizing task was removed and replaced with trials of the reasoning and typing task to keep the total number of *N* = 60 trials the same. For the current analysis, we, therefore, used data only from the reasoning and the typing tasks excluding the memorizing task from all further analyses. Driving performance data, including lateral car position and velocity of the participants’ car, were recorded at 10 Hz. Preprocessing is detailed below (see “Driving Behavior” section). Performance of the additional tasks (reasoning and typing) was not evaluated in this study as we were only interested in driving behavior.

#### Cognitive Functions

Fluid cognitive functions were assessed by computer-based tests adapted from literature, all of which were programmed in E-Prime 2.0 (Psychology Software Tools, Pittsburgh, PA, USA). Each test took about 10 min. Stimuli were presented on a 24′′ monitor (1,920 × 1,080 screen resolution). All stimuli were black and presented on a white screen background. Standardized instructions were displayed first, followed by up to three practice runs. Response feedback was provided after practice trials, but not after registered trials. All tests comprised six blocks of stimuli that were separated by inter-block breaks of 5 s (20 s after block 3). The response-stimulus interval was 800–1,200 ms; if there was no response on the preceding trial, the response-stimulus interval started after 2,000 ms. Participants responded by pressing the “X” or “M” key on a German keyboard with their left and right index finger. They were instructed to respond as fast and as accurately as possible. The reaction time of correct responses (RT) and the percentage of correct responses across all presented stimuli (ACC) were analyzed.

A visuospatial *n-back test* (2-back) was used to measure *updating of working memory* (“updating”; Schmiedek et al., 2009). Each block comprised a total of 19 dots that were sequentially presented for 500 ms in one field of a black 4 × 4 grid. Participants were asked to press the “M” key if the current dot appeared at the identical position as the dot two trials before (target), and to press the “X” key if the dot appeared at a different position (non-target). The first two stimuli of each trial were discarded from the analysis.

The *Simon test* was administered to measure *inhibition* (Simon and Wolf, 1963; Simon and Rudell, 1967). Each block included a total of 32 trials of left- or rightward pointing arrows that were sequentially presented for 500 ms to the left or right of a centered fixation cross. For 50% of the trials, the direction and position of the arrow were congruent (e.g., rightward arrow on the right side); for the other 50% of trials, they were incongruent (e.g., rightward arrow on the left side). Participants were instructed to press the left key (“X”) for leftward pointing arrows, and the right key (“M”) for rightward pointing arrows.

A spatial *task* s*witching test* was used to measure *shifting* (modified from Kray and Lindenberger, 2000). Each block included a total of 17 trials that were sequentially presented in the middle of the screen for 1,500 ms. Each stimulus was either a circle or a rectangle and was either small or big. Participants had to respond to either the size (A) or the form (B) of the stimuli in the order AA-BB-AA-BB-AA-BB-AA-BB-A. Participants had to press the “X” key for small or circular stimuli, and the “M” key for big or rectangular stimuli. The first stimulus of each trial was not analyzed.

*Cognitive processing speed* was derived from the *congruent Simon test* condition. The congruent Simon test condition affords only simple reactions to the pointing direction (left/right) of arrows involving only little cognitive demand and therefore reflects simple cognitive processing speed.

#### Cardiovascular Fitness

Spiroergometry (ZAN600 CPET, nSpire Health, Oberthulba, Germany) on a stationary bicycle (Lode Corival cpet, Groningen, the Netherlands) was used to assess *cardiovascular fitness*. Participants were asked to avoid intake of caffeine and alcohol for 12 h and any vigorous physical activities for 24 h before testing. A ramp protocol was applied to test for submaximal exhaustion (Niemann et al., 2016; Hübner et al., 2019; Stute et al., 2020). Participants were instructed to maintain a cycling frequency between 60 and 80 revolutions per minute. In project phase I, participants started at 30 W initial load that increased progressively by 10 W (female) or 15 W (male) per minute. Participants of project phase II started at 10 W (female) or 20 W (male) initial load that increased progressively by 15 W (female) or 20 W (male) per min. Ramp protocols were preceded by a 3 min resting period and followed by a 5 min cool-down (1 min initial load, then no load). In total, protocols lasted about 15–20 min. Electrocardiography [ECG, recorded with a 10-lead ECG fully digital stress system; Kiss, GE Healthcare, Munich, Germany), breath-by-breath respiration (oxygen uptake (VO_2_), carbon dioxide output (VCO_2_)], heart rate, blood pressure (every 2 min), and wattage were continuously assessed. Further, the respiratory exchange ratio (VCO_2_/VO_2_) was simultaneously determined. A Borg’s ‘rate of perceived exertion’ scale (6–20: “very easy” – “very difficult”) was administered every 2 min to ask for perceived exertion during cycling. The protocol was stopped when participant’s respiratory exchange ratio remained > 1.05 for at least 30 s or exceeded 1.10, upon volitional fatigue, or occurrence of risk factors (i.e., heart rate, HR > about 220-age, blood pressure >230/115 mmHg, dizziness, cardiac arrhythmia, or other abnormalities). The outcome measure was peak oxygen consumption (VO_2_ peak), which has been proposed as a sufficient indicator of cardiovascular fitness (Rankovic et al., 2010).

Spiroergometry was supervised by an experienced sports scientist. In project phase I, the ramp protocol was preceded by an additional, less demanding, alternating 30 W/80 W protocol that was performed for approximately 10–15 min. Due to technical issues, five participants of project phase II were tested using a different spiroergometry device (Oxycon Pro, Erich Jaeger GmbH, Hoechberg, Germany); data, however, were comparable to the other participants following visual inspection and therefore were handled accordingly.

#### Motor Coordinative Fitness

Motor coordinative fitness was assessed with a battery of three standardized tests for different domains of motor coordinative fitness (Voelcker-Rehage et al., 2010). Before each test, participants were shortly familiarized with the procedure and were controlled for correct performance. Time was kept using a stopwatch. *The Purdue Pegboard Test* (Purdue Pegboard test, model 32020, Lafayette Instruments, Lafayette, IN, USA) was administered to measure *bimanual dexterity* (Tiffin and Asher, 1948; Tiffin et al., 1985). Participants were asked to plug as many metal pegs as possible into two parallel rows (maximum 25 holes) of the pegboard with both hands simultaneously, from top to bottom, and hole by hole. Three runs were performed, each timed at 30 s. The outcome measure was the number of holes with correctly placed pegs, averaged across the three runs. *The Feet Tapping Test* was used to measure *psychomotor speed* (Voelcker-Rehage and Wiertz, 2003; Voelcker-Rehage et al., 2010). Participants were seated on a stationary chair and instructed to tap with both feet simultaneously back and forth across a mid-sagittal line on the floor for a duration of 20 s. They were instructed to move both feet completely across the line, with both soles flat on the floor. The outcome measure was the number of correct crossings, as assessed with a hand clicker. The better of two runs was selected for analysis. The* One-Leg Standing* *Test* with eyes open and eyes closed was performed to assess *static balance* (Ekdahl et al., 1989). Participants looked straight ahead and stood on one leg, while slightly flexing the other leg, for a maximum of 20 s (self-initiated). Eight runs were performed, four runs with eyes open and then four runs with eyes closed (two runs each per leg). Time was stopped when participants put down their lifted foot, pressed together their legs, hopped, or opened their eyes during closed eyes balancing. Due to a very distinct ceiling effect for eyes open balancing, only eyes closed balancing was analyzed. The outcome measure was the standing duration, averaged across all four runs of eyes closed balancing (Michikawa et al., 2009).

### Procedure

Before the first day of testing, all eligible participants received general information about the project, informed consent forms, and a questionnaire on demographics, handedness, driving, physical, and social activities, and health status. Testing was distributed across 4 days in project phase I and 2 days in project phase II where fewer tests were administered. Total testing time, including test instructions and small breaks, was 4 (project phase II) to 8 h (project phase I) per participant. For both project phases, the same measures of cardiovascular and motor coordinative fitness, fluid cognitive functions, and driving were used (for an overview cf. Table 2). The order of tests followed different pseudorandomized schedules, to take serial-order effects into account. Differences between measures were accounted for by standardizing test scores per project phase.

**Table 2:** Overview of all tests and variables used for statistical analysis.

Variable	Test	Raw measures	Performance indicator
Cardiovascular Fitness (IV_exo_)	Spiroergometry	Oxygen uptake	VO_2_ peak
Motor Fitness (IV_exo_)	Purdue Pegboard Test (1), One-Legged Stand Test with closed eyes (2), Feet Tapping Test (3)	Number of correct pegs within 30 s (1), Average standing duration (max. 20 s; 2), Number of correct crossings/taps within 20 s (3)	Composite score (mean of the *z*-standardized scores)
Updating (IV_endo_)	*n*-Back Test	RT, ACC	BIS
Cognitive Processing Speed (IV_endo_)	Simon Test	RT (only congruent)	RT
Inhibition (IV_endo_)	Simon Test	ΔRT, ΔACC	BIS
Switching (IV_endo_)	Task Switching Test	ΔRT, ΔACC	BIS
Speed Control (DV)	Virtual Driving Scenario	Car velocity	Mean velocity during additional task performance
Lane Keeping (DV)	Virtual Driving Scenario	Lateral car position on the lane	SD of the lateral position during additional task performance

*Note. Summary of the variables used for path analysis (for further details please refer to section “Measures” and “Data Analysis”). ACC, accuracy (in %); ΔACC, |congruent − incongruent| (in ms); BIS, balance integration score (z-scaled); DV, dependent variable; endo, endogenous; exo, exogeneous; IV, independent variable; RT, reaction time (in ms); ΔRT, |congruent − incongruent| (in ms); SD, standard deviation; VO_2_ peak, peak oxygen uptake (in l/min)*.

### Data Analysis

Raw data were preprocessed with E-Prime 2.0 (cognitive functions) and R Studio v1.1.463 (R Core Team, 2020; cardiovascular and motor coordinative fitness, driving behavior) using the same routines for participants of both project phases.

#### Driving Behavior

Driving behavior under dual-task conditions was analyzed for time segments of 0–10 s after the onset of an additional task. Data from our previous studies suggested that the effects of additional tasks persist for about 10 s after task onset. In addition, this time range closely corresponded to the minimal inter-stimulus interval (*M* = 17.75, *SD* = 4.55) across all tasks in both project phases. Therefore, this also was the longest interval without any overlap between additional tasks. Outcome measures were the standard deviation of the mean lateral position of the participant’s car on the lane (Latpos SD, in m) and the average velocity of the participant’s car (Velocity M, in m/s). Both measures are commonly used in car driving research (Wechsler et al., 2018; Depestele et al., 2020; Stojan and Voelcker-Rehage, 2021). They were computed for all reasoning and typing trials. Outliers were excluded using the ±3.29 SD criterion for each participant (Tabachnick and Fidell, 2007). Data were then averaged across all trials, including task types (reasoning, typing) and presentation modalities (visual, auditory). Missing data (Latpos SD: *n* = 3, Velocity M: *n* = 3) were imputed by regression imputation as part of the path analysis (see “Statistical Approach” section). As described above, the performance (RT, ACC) of the additional tasks while driving was not analyzed separately (for further information on the additional tasks see Wechsler et al., 2018; Stojan and Voelcker-Rehage, 2021).

#### Cognitive Functions

In the first step, RTs (ms) were removed if RT < 80 ms or RT >1,300 ms. Second, remaining RTs were outlier corrected using the ± 3.29 SD criterion for each participant. Updating performance was quantified as the mean RT and ACC across target and non-target trials. Inhibition performance was calculated from the RT difference between congruent and incongruent trials, and from the corresponding ACC difference. Shifting performance was computed as the difference value of switch trials (i.e., AB and BA) and repeat trials (i.e., AA and BB), again separately for RT and ACC. Finally, cognitive processing speed was determined as the mean RT of all congruent responses; ACC for cognitive processing speed was not considered, as it was close to 100% for congruent trials in all participants. For all cognitive tests, participants with ACC <55% were considered random performers, and their performance on that particular test was treated as missing data. Missing data (in total: updating: *n* = 14, inhibition: *n* = 1, shifting: *n* = 22, cognitive processing speed: *n* = 0) were imputed by regression imputation as part of the path analysis (see “Statistical Approach” section).

To account for a speed-accuracy tradeoff in updating, inhibition, and shifting tests, RT and ACC scores were converted into a standardized performance index, the balance integration score (BIS), using.

(1) BIS=z(RT)−z(AAC)

BIS is considered to best account for the speed-accuracy trade-off compared to other approaches, as it puts equal weights on RT and ACC (Liesefeld and Janczyk, 2019). Finally, the inverse of BIS was calculated to facilitate interpretation, with higher scores indicating better performance.

#### Cardiovascular Fitness

VO_2_ peak was determined by applying a moving average filter (lag 20, two-sided to avoid phase distortion) to the VO_2_ continuous time series data in order to improve the subsequent peak detection by accounting for the typical breath by breath fluctuations. VO_2_ peak was identified as the maximum value in the range of the highest complete performance level (wattage) achieved by the participant. The accuracy of the peak detection was visually inspected for every participant. In addition, we determined whether participants reached their submaximal performance level considering a VO_2_ peak >1.5 l/min and a respiratory exchange ratio coefficient (>1.0) at their highest wattage level. If participants did not meet these criteria (e.g., due to lack of motivation), the VO_2_ peak was specified as missing. Missing data (*n* = 23) were imputed by regression imputation as part of the path analysis (see “Statistical Approach” section).

#### Motor Coordinative Fitness

No outlier correction was applied to the performance scores described above. For each participant, an overall index of motor coordinative fitness was calculated as the mean of the z-standardized scores for psychomotor speed, static balance, and bimanual dexterity (Voelcker-Rehage et al., 2010; Niemann et al., 2016). Hence, all three measures were equally weighted. Missing data (*n* = 3) were imputed by regression imputation as part of the path analysis.

### Statistical Approach

Path analysis was used to test the hypothesized linear relationships of our proposed model (Figure 1). Path analysis often is referred to as a special case of structural equation modeling (Jöreskog, 1970; Byrne, 2010; Tarka, 2018). It builds upon multiple regression and is applied to investigate complex relationships between dependent (endogenous variables) and independent variables (exogenous variables; Wright, 1934; Stage et al., 2004). As the main advantage of path analysis over basic multiple regression, it allows an estimation not only of direct effects between variables, but also an estimation of indirect effects mediated *via* one or multiple intermediate dependent variables.

Figure 1 illustrates our proposed model structure. Primary dependent variables were driving performance (Latpos SD and Velocity M). Intermediate dependent variables were cognitive functions (updating, inhibition, shifting, and cognitive processing speed). Independent variables were fitness domains (cardiovascular fitness and motor coordinative fitness). According to our hypothesized theoretical model, direct pathways were considered between driving performance and cognitive functions, as well as between cognitive functions and fitness domains. As described above, we hypothesized indirect but no direct pathways between fitness domains and driving performance. Direct and indirect pathways are displayed in Figure 1. Direct effects are depicted as solid lines. Indirect effects (labeled as ‘IE’) are depicted as solid lines passing the intermediate structure of cognitive functions as dotted lines.

The linear relationship between variables was tested simultaneously using path analysis with SPSS (version 26; IBM Corp., Armonk, NY, USA) AMOS 26.0 (Arbuckle, 2014). A missing value analysis was performed (Little’s MCAR test; Little, 1988), indicating that data were missing completely at random (*χ*^2^ = 73.33, DF = 75, *p* = 0.53). Missing values (7.19% of all data) were handled through regression imputation (Acock, 2005; Madley-Dowd et al., 2019), by which missing data points are determined based on participants with similar values. Fit of the proposed model was assessed by comparative fit index (CFI), Tucker-Lewis Index (TLI), the standardized root mean square residual (SRMR), and root mean square error of approximation (RMSEA). Values exceeding 0.90 for the CFI and TLI, and values less than 0.09 and 0.08 for the SRMSR and RMSEA, respectively, are indicative of strong model fit (Marsh et al., 2004).

## Results

The proposed model (see Figure 1) gave a strong fit to our experimental data according to all criteria adopted, *χ*^2^ = 11.30, *df* = 12, *p* = 0.418; CFI = 0.98; TLI = 0.96; RMSEA = 0.015, 95% CI RMSEA = 0.000, 0.098; SRMR = 0.051. Standardized beta coefficients for the model pathways are presented in Figure 1.

*Main effects—Cognition on Driving*: Updating (*β* = 0.19, *p* = 0.035), but not Cognitive Processing Speed (*β* = 0.11, *p* = 0.216), Inhibition (*β* = 0.07, *p* = 0.440), and Shifting (*β* = 0.11, *p* = 0.200) predicted Latpos SD. Inhibition (*β* = 0.23, *p* = 0.009), but not Updating (*β* = −0.04, *p* = 0.665), Cognitive Processing Speed (*β* = 0.05, *p* = 0.599), or Shifting (*β* = −0.01, *p* = 0.882) predicted Velocity M.

*Main effects—Physical Fitness on Cognition*: Cardiovascular Fitness predicted Cognitive Processing Speed (*β* = 0.18, *p* = 0.039), but not Updating (*β* = −0.06, *p* = 0.499), Shifting (*β* = −0.06, *p* = 0.536), or Inhibition (*β* = 0.14, *p* = 0.123). Motor Coordinative Fitness predicted Updating (*β* = 0.25, *p* = 0.004) and Cognitive Processing Speed at trend level (*β* = 0.17, *p* = 0.053), but not Inhibition (*β* = 0.00, *p* = 0.962) and Shifting (*β* = 0.00, *p* = 0.982).

*Indirect effects—Physical Fitness on Driving*: Cardiovascular Fitness showed a trend-level indirect effect on Velocity M (*β* = 0.04, 95% CI 0.001, 0.097, *p* = 0.096), but no indirect effect on Latpos SD (*β* = 0.01, 95% CI −0.058, 0.079, *p* = 0.815). Motor Coordinative Fitness yielded a significant indirect effect on Latpos SD (*β* = 0.07, 95% CI 0.010, 0.133, *p* = 0.036), but no effect on Velocity M (*β* = 0.00, 95% CI −0.056, 0.050, *p* = 0.961).

## Discussion

In this study, we investigated whether physical fitness is positively associated with driving performance under dual-task conditions by mediation through cognitive functioning in healthy older adults. We hypothesized an indirect pathway by which two domains of physical fitness, i.e., cardiovascular and motor coordinative fitness, facilitate cognitive functioning (i.e., updating, shifting, inhibition, and cognitive processing speed) and thus driving (i.e., lane keeping, speed control) while performing cognitively demanding tasks. In accordance with extant literature, we observed that driving behavior and cardiovascular and motor coordinative fitness were directly related to specific, but different, fluid cognitive functions. As a result of these direct relationships and in accordance with our hypothesis, we found that motor coordinative fitness demonstrated a significant indirect effect on lane keeping, but not on speed control. Cardiovascular fitness, in contrast, showed a trend-level indirect effect on speed control, but not on lane keeping. Hence, being physically fit in older age seems to promote cognitive functions that are positively associated with driving and may therefore benefit car driving performance in older adults.

### Direct Relationships Between Cognition and Driving Under Dual-Task Conditions

Cognitive functions were related to driving behavior in cognitively demanding conditions in the current study. Updating was associated with lane keeping, while inhibition was related to speed control. Speed control requires attentional and anticipatory control mechanisms to avoid collisions with a lead car, specifically when engaging in an additional, distracting task during driving. Attentional and anticipatory processes, in turn, are related to inhibition (Diamond, 2013; Elchlepp et al., 2016; Grandjean et al., 2019). Hence, impaired inhibition in older drivers might be associated with inferior attentional and anticipatory processes leading to poorer speed control (Anstey and Wood, 2011; Fofanova and Vollrath, 2011; Hahn et al., 2011; Karthaus and Falkenstein, 2016). This might become even more visible in a distracting or cognitively demanding driving condition. Lane keeping, in turn, requires sustained situational awareness and continuous monitoring and updating of visuospatial information of the environment (e.g., road winding; Papantoniou et al., 2017; Nilsson et al., 2020). Performing an additional task during driving typically increases cognitive load, thus limiting available cognitive resources. Additional tasks may cause interference effects on lane keeping due to limited updating capacity in older adults (i.e., capacity interference; Son et al., 2011; Pettigrew and Martin, 2016; Nilsson et al., 2020). Furthermore, when additional tasks require similar cognitive and perceptual resources, interference effects (i.e., structural interference) are typically more pronounced (Heuer, 1993; Liu and Ou, 2011; Stelzel and Schubert, 2011; Engstrom et al., 2017; Leone et al., 2017; Stelzel et al., 2017; Wechsler et al., 2018; Bohle et al., 2019; Perlman et al., 2019). Individuals with lower updating capacity hence show higher interference effects on lane keeping during driving when engaging in an additional task.

The observed relationships between cognitive functions and driving behavior, however, were quite specific and rather small. A recent systematic review also found that cognitive functions associated with driving behavior in younger and older adults were quite inconsistent across studies (Depestele et al., 2020). For older adults, some studies demonstrated such a relationship (Andrews and Westerman, 2012), while others did not (Park et al., 2011; Chen et al., 2013; Eudave et al., 2018). Varying relationships between cognitive functions and driving parameters might be attributed to different driving settings, task designs, and outcome measures (Park et al., 2011; Bunce et al., 2012; Eudave et al., 2018). In addition, driving experience may also influence cognitive demands during driving. Higher driving experience typically leads to generally less cognitive involvement during driving due to higher automatization (McKenna and Farrand, 1999; Harada et al., 2007; Charlton and Starkey, 2011). Assuming that our samples of older adults were quite experienced drivers (based on our inclusion criteria), cognitive demand during driving may have been reduced compared to more inexperienced drivers, such as younger or middle-aged adults or less experienced older drivers (Mourant and Rockwell, 1972; Lee, 2008; Emerson et al., 2012). Furthermore, experienced drivers may perform additional tasks more frequently while driving or use compensation strategies more efficiently, resulting in some degree of automation in these tasks as well, thus leading to less involvement of cognitive functions (Divekar et al., 2012; Klauer et al., 2014; Karthaus and Falkenstein, 2016; Pope et al., 2017). However, further research is needed to address additional factors influencing cognitive mechanisms associated with driving behavior, in particular for dual-task driving scenarios (Depestele et al., 2020).

### Direct Relationships Between Fitness and Cognition

We observed direct relationships of cardiovascular and motor coordinative fitness with specific fluid cognitive functions. Cardiovascular fitness was related to cognitive processing speed only, whereas motor coordinative fitness was associated with cognitive processing speed and updating. This is at least partly in line with previous experimental findings and review studies. Results typically vary between studies, potentially as a result of, for example, different study designs, sample characteristics, or cognitive measures (Voelcker-Rehage et al., 2010; Hötting and Röder, 2013; Voelcker-Rehage and Niemann, 2013; Bherer, 2015; Dupuy et al., 2015; Kawagoe et al., 2017; Mekari et al., 2019). The effects of motor coordinative fitness on cognitive functions seem to be more pronounced than for cardiovascular fitness (Angevaren et al., 2008; Johann et al., 2016; Ludyga et al., 2020). This is in line with a current meta-analysis indicating higher benefits of exercise on cognitive functioning after motor coordinative exercise compared with other exercise types (Ludyga et al., 2020). Even though motor coordinative fitness has received far less scientific attention than cardiovascular fitness, the underlying mechanisms of cognitive benefits are discussed to be quite distinct (Cotman and Berchtold, 2007; Forte et al., 2013; Voelcker-Rehage and Niemann, 2013; Netz, 2019). Cardiovascular fitness is assumed to mainly induce changes in brain metabolism associated with improved cerebral blood flow and vascularization (Sonntag et al., 2007; Smith et al., 2010; Dupuy et al., 2015; Tarumi and Zhang, 2018; Kleinloog et al., 2019). Motor coordinative fitness, to the contrary, seems to induce fewer metabolic changes, but be related to functional changes such as higher synaptic density and network connectivity (Black et al., 1990; Lee et al., 2013; Cai et al., 2014; Demirakca et al., 2016). These brain physiological changes appear to be rather global than specific for both types of fitness, even though effects seem to be most pronounced in frontal areas (Voelcker-Rehage and Niemann, 2013; Reuter-Lorenz and Park, 2014; Bherer, 2015). As a result, the differential mechanisms of cardiovascular and motor coordinative fitness might favor effects on specific cognitive functions such as executive functions, which are associated with mutual but also unique neural correlates (Miyake and Friedman, 2012; Friedman and Miyake, 2017). Also, cognitive processing speed was positively associated with both cardiovascular and motor coordinative fitness. Cognitive processing speed reflects a rather global and relatively region-unspecific, lower-level cognitive function and has been suggested previously to benefit more from physical activity/fitness than other cognitive functions (Salthouse, 1996, 2009; Chang et al., 2010; Albinet et al., 2012; Quigley et al., 2020) as the effects of physical activity/fitness are assumed to be quite global as well (Dustman et al., 1984; Hillman et al., 2002; Angevaren et al., 2008). Among the included executive functions, only updating was related to higher (cardiovascular) fitness. Updating has been associated with frontoparietal brain regions specifically (Smith and Jonides, 1997; Wager and Smith, 2003). The neural overlap with exercise-induced changes hence might be higher for updating than for inhibition or shifting (Collette et al., 2005), even though those functions also have been related to frontal but also parietal brain areas (Bissonette et al., 2013; Zhang et al., 2017). In order to understand the relationship between certain cognitive functions and specific physical fitness parameters, systematic investigations are required that are beyond the scope of the current article.

### Indirect Relationships Between Fitness and Driving Under Dual-Task Conditions

Most importantly, both cardiovascular and motor coordinative fitness were found to yield an indirect effect on driving behavior during additional task performance. Those indirect relationships were based on the described direct associations of cardiovascular and motor coordinative fitness with cognitive functions, and the direct associations of cognitive functions with driving behavior. Even though the magnitude of those direct effects differed largely, the sum of them led to the hypothesized indirect relationships. Interestingly, cardiovascular and motor coordinative fitness were positively related to distinct driving parameters. Motor coordinative fitness was associated with lane keeping, while cardiovascular fitness was associated with speed control. As discussed above, cardiovascular and motor coordinative fitness are thought to promote cognitive functioning *via* distinct neurobiological mechanisms, thus, eventually leading to differential effects on specific cognitive functions (Black et al., 1990; Sonntag et al., 2007; Lee et al., 2013; Cai et al., 2014). Those cognitive functions, again, are differentially associated with specific driving behavior parameters (Depestele et al., 2020). These distinct relationships could explain the observed differential associations between fitness domains and driving behavior parameters. However, it should be noted that, overall, the observed effects (direct and indirect) were rather small (indirect effects: *ß* = 0.00–0.07). Nevertheless, even small effects might be of practical relevance and could be associated with a reduced risk for accidents during everyday car driving in older age. As mentioned above, the observed effects might be even more pronounced in inexperienced drivers as they rely more on cognitive control during driving and additional task performance, and thus may show greater benefits from higher physical fitness. In addition, our samples of older individuals were very fit, both physically and cognitively, thus limiting the available variance to explain the hypothesized indirect effects. We assume that the indirect effects of physical fitness on driving behavior would have been even more pronounced in a sample with a broader range of physical fitness levels as usually observed for the direct effects on cognitive functions (Voelcker-Rehage et al., 2010; Voelcker-Rehage and Niemann, 2013; Bamidis et al., 2015). Furthermore, considering that several cognitive functions relevant for driving (Karthaus and Falkenstein, 2016; Depestele et al., 2020) have not been assessed in this study (e.g., visuospatial skill or attention), the observed indirect effects might rather underestimate the real effects of fitness on driving behavior. In conclusion, our findings indicate that maintaining a high level of cardiovascular and/or motor coordinative fitness may facilitate real-car driving in older adults, thus promoting mobility and an independent lifestyle in higher ages.

## Strength and Limitations

In this study, we used an ecologically valid, virtual driving scenario with realistic additional tasks that are frequently performed during everyday driving. We believe that such a task setting is favorable over less realistic settings (e.g., passively observing driving scenes or studies using abstract laboratory tasks) to infer potential effects on real-life behavior. The first limitation of our study, however, regards the above-mentioned lack of measures of additional cognitive functions associated with driving behavior. As a result, the observed indirect effects are likely to be underestimated and should be complemented by studies using additional measures for cognitive functions. Similarly, the second limitation of this study is that we did not address the association of other physical domains, such as physical strength, on cognitive functions and driving behavior. Similar to cardiovascular and motor coordinative fitness, physical strength seems to be associated with cognitive functioning in older adults (Chang et al., 2011; Ludyga et al., 2020). Therefore, physical strength may also yield indirect benefits for driving behavior *via* improved cognitive functioning. A third limitation concerns the analysis of fixed sections of driving performance after task presentation. The effects of additional tasks on driving behavior are likely to vary in their duration between different types of tasks, but also between participants. Thus, those effects might be analyzed in more detail using adapted time spans rather than a uniform time window of 10 s. For example, time windows might be defined for each task and presentation modality separately, depending on the persistence of their individual effects on driving behavior. However, based on previous findings the effects of additional tasks on driving seem to be most distinct within the first seconds after task onset (Strayer and Drew, 2004). Hence, we chose to analyze driving performance closely after task onset, and we utilized a uniform time window as we were not interested in the specific effects of different tasks or presentation modalities in the current study.

## Conclusion

For the first time, we demonstrated that both cardiovascular and motor coordinative fitness are indirectly associated with driving in presence of additional cognitive demands in healthy older persons. Specifically, motor coordinative fitness was associated with cognitive functions that are involved during lane keeping, while cardiovascular fitness was associated with cognitive functions that are involved during speed control. By way of these associations, being physically active/fit may reduce the risk of accidents in cognitively challenging driving situations. At a more general level, our findings demonstrate that fitness may promote complex real-life behavior in older persons by supporting cognitive functioning. These findings are in accordance with the view that cognitive benefits associated with higher cardiovascular and motor coordinative fitness indeed facilitate complex cognitive-motor behaviors of everyday life. Future research should include additional fitness parameters (e.g., physical strength), cognitive measures (e.g., attention, visuospatial skills), driving parameters (e.g., number of crashes, braking performance), or may investigate other real-life behaviors (e.g., wayfinding, “walking while talking”) in addition.

## Data Availability Statement

The raw data supporting the conclusions of this article will be made available by the authors, without undue reservation. Requests to access the datasets should be directed to CV-R (claudia.voelcker-rehage@uni-muenster.de).

## Ethics Statement

The studies involving human participants were reviewed and approved by the ethics committee of the German Sport University, Cologne, Germany (Nr.: 27/2015) and the ethics committee of the Chemnitz University of Technology, Germany (Nr.: V-280-17-CVR-Multitasking-29062018). The patients/participants provided their written informed consent to participate in this study.

## Author Contributions

RS, NK, OB, and CV-R were responsible for conceptualization and methodology of the research question and data analyses of the current manuscript. RS and NH were responsible for investigation. RS carried out software for data preprocessing, data curation and visualization. Formal analysis was performed by RS and NK. RS and NK wrote the original draft. NK, OB, and CV-R critically reviewed and edited the manuscript. Project administration (overall project) was carried out by OB and CV-R, project administration (research question reported here) was carried out by RS and NK. Funding acquisition, resources, and supervision were provided by OB and CV-R. All authors contributed to the article and approved the submitted version.

## Conflict of Interest

The authors declare that the research was conducted in the absence of any commercial or financial relationships that could be construed as a potential conflict of interest.
